# Prevalence of Aspirin or Clopidogrel Pharmacological Resistance in Ischemic Stroke: A Step Toward Precision Medicine

**DOI:** 10.1111/cns.70343

**Published:** 2025-03-18

**Authors:** Samantha Cencer, Laurel Packard, Alan Davis, Asad Ahrar, Malgorzata Miller, Nadeem Khan, Nabil Wees, Jiangyong Min

**Affiliations:** ^1^ Department of Neurosciences and Comprehensive Stroke Center Corewell Health West Grand Rapids Michigan USA; ^2^ Department of Neurology Michigan State University College of Human Medicine Grand Rapids Michigan USA; ^3^ Department of Statistics and Probability Michigan State University Lansing Michigan USA

**Keywords:** aspirin, clopidogrel, ischemic stroke, pharmacological resistance

## Abstract

**Background and Objectives:**

Currently, there is not sufficient data regarding the prevalence of resistance or inadequate platelet function inhibition with the use of antiplatelet therapy in patients with noncardioembolic stroke. This study was designed to evaluate the prevalence of antiplatelet inactivity to aspirin and clopidogrel in the setting of chronic use and presentation with primary or recurrent stroke.

**Methods:**

Patients who were taking aspirin, clopidogrel, or both at the time of presentation for stroke were selected in this study. Those with confirmed stroke on MRI or clinically determined TIA and age > 18 years were included. A standard laboratory test, VerifyNow aspirin or P2Y12 assay, was utilized to assess the responsiveness to the platelet inhibitors. A total of 158 patients were identified, 52 presenting with primary stroke and 106 with recurrent stroke. Data were analyzed using chi‐squared or Fisher's exact as well as *t*‐test analysis.

**Results:**

Of the primary stroke population, 4% of patients demonstrated resistance to aspirin and 30% to clopidogrel. Of the patients presenting with recurrent stroke, 13% demonstrated resistance to aspirin and 38% to clopidogrel. The data also suggest increased resistance to aspirin and clopidogrel in Caucasians compared to minorities, with 11% versus 8% in regard to aspirin and 33% versus 17% to clopidogrel. Additionally, this study demonstrated 17% resistance to aspirin in males compared to 4% in females and 13% in males compared to 36% in females, respectively, regarding resistance to clopidogrel. No difference in inactivity to either aspirin or clopidogrel was detected between patients with stroke mechanisms of small or large vessel disease.

**Conclusions:**

The present result demonstrated that a sizeable portion of the population has inefficacious activity in the setting of specific antiplatelet agents. Additionally, sex and ethnicity differences in responsiveness to aspirin or clopidogrel have been noted. Determining a patient's response to medications could provide opportunities to individualize treatment and better prevent future strokes. Further studies of a larger scale are indeed needed to apply this information to pursue individualized treatment.

## Graphical abstract

The present result suggests that a sizeable portion of the population has inefficacious activity in the setting of certain antiplatelet agents. Additionally, sex and ethnicity differences in responsiveness to aspirin or clopidogrel have been noted.

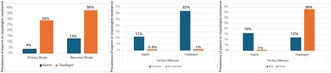

## 1 Introduction

Acute ischemic stroke is a pathologic process affecting 795,000 patients annually in the United States, with recurrent events occurring in approximately 185,000 of these patients [1]. Despite advancements in medical management, stroke remains the fourth leading cause of death [2], following cardiovascular disease, cancer, and accidents. It is also the leading cause of disability [3], imposing a significant financial and social burden on families and society. Aspirin and clopidogrel are popular medicines routinely used for secondary cardiovascular or cerebrovascular prevention. Aspirin is a readily available medication that reduces the risk of cardiovascular or cerebrovascular disease by about 25% in patients with atherosclerotic disease [4]. More than 3 million people annually in the United States alone receive the antiplatelet drug clopidogrel after cardiac stenting for acute coronary syndrome [5]. Clopidogrel was approved by the Food and Drug Administration (FDA) in 1997 to reduce atherosclerotic events. It was known to be a prodrug, but it was not until 2016 that CYP2C19 was identified as the bioactivating enzyme [6]. Dual antiplatelet therapy (DAPT) with aspirin and clopidogrel is recommended by the American Stroke Association (ASA) for intracranial stenosis [7] and high‐risk transient ischemic attack (TIA) or minor ischemic stroke [8]. No doubt aspirin and clopidogrel provide significant clinical benefits in patients with cerebrovascular disease. However, given the complexity of platelet activation, it is unsurprising that aspirin and clopidogrel prevent only a certain proportion of vascular events in the presence of tightly controlled common risk factors. Of late, the terms aspirin and clopidogrel “resistance” have entered the physicians' view and infer a lack of therapeutic response due to minimal inhibition of platelet function in patients taking aspirin or patients with poor metabolizer phenotype variation who receive clopidogrel. It has been learned that single nucleotide polymorphisms of the CYP2C19 gene could result in altered metabolism of this antiplatelet prodrug [9]. Lyerly and colleagues [10] advocated pursuing platelet function studies to understand better an individual's ability to respond to clopidogrel in patients who are at high risk of recurrent stroke or who have experienced a recurrent cerebrovascular event, especially in the new era of precision medicine.

A few studies have been conducted over the years to create guidelines focused on the choice of agents, timing, duration of therapies, and the efficacy of individual treatments. Still, there are relatively limited data regarding how to approach resistance or hyporesponsiveness to these agents. A pillar of stroke management for years has included the use of antiplatelet agents, in large part with aspirin and clopidogrel. A prior study has suggested resistance to these agents in a statistically significant portion of the population through the use of multiple electrode aggregometry testing [11]. Questions were raised to categorize further the prevalence of resistance in different subsets of the population where minimal data remain. This study provides additional information on ultimately guiding individualized treatment plans and increasing the efficacy of secondary stroke prevention.

## 2 Methods

This single‐center study included 158 patients with primary or secondary stroke admitted at Corewell Health in Grand Rapids and Michigan State University, Michigan. The retrospective cross‐sectional study was approved by our institutional review board (IRB). Inclusion criteria involved patients with primary or recurrent ischemic stroke confirmed on magnetic resonance imaging (MRI) or clinically determined (transient ischemic attack [TIA]) (≥ 18 years of age) who were taking aspirin, clopidogrel, or aspirin and clopidogrel at the time of presentation. Patients with determined stroke mimics, strokes due to mechanisms requiring anticoagulation, and those with medication noncompliance were excluded. A standard laboratory test using VerifyNow aspirin [12] or VerifyNow‐P2Y12 assay [13] was utilized to assess the level of platelet inactivation with the use of aspirin and clopidogrel with the primary endpoint of determined inadequate platelet inactivation response on these assays. Aspirin reactivity unit (ARU) value below 550 is indicative of effective aspirin inhibition of platelet activity. ARU value of 550 or above is indicative of ineffective aspirin inhibition of platelet activity. VerifyNow‐P2Y12 assay result is reported in platelet reactivity unit (PRU). Patients on clopidogrel therapy with PRU value at or below 180 are considered adequate responders. Patients with PRU value at or above 208 are considered ineffective platelet clopidogrel inhibition of platelet activity. Patients with PRU values between 108 and 208 had VerifyNow‐P2Y12 repeated in 12–24 h. Demographic data were also collected, including age, sex, ethnicity, stroke risk factors, and data regarding the location of stroke and determined etiology. Data were then analyzed using chi‐squared or Fisher's exact and *t*‐test analysis. Statistical significance was defined at *p* < 0.05.

## 3 Results

A total of 158 patients were identified, 52 presenting with primary stroke and 106 with recurrent stroke. The average age of patients with primary stroke was 68 years, with 92% being Caucasian and 57% being male. Twenty patients were smokers with an average body mass index (BMI) of 29, low‐density lipoprotein (LDL) of 94, and hemoglobin A1c (HbA1c) of 6.4. Twenty‐seven patients had a history of hypertension (HTN), 15 with coronary artery disease (CAD), and 6 with peripheral vascular disease (PVD). The average National Institutes of Health Stroke Scale (NIHSS) at admission was 3.5 and 2.4 at discharge. Thirty‐two patients were on aspirin, eight on clopidogrel, and 12 were on both agents at presentation. Classification of stroke was divided into 14 patients with small vessel disease, 15 with large vessel disease, 10 with cardiogenic origin, and 13 cryptogenic. Regarding patients with recurrent stroke, the average age of patients was 67 years, with 91% being Caucasian and 59% being male. Forty patients were smokers with an average BMI of 31, LDL 82, and HbA1c 6.3. Fifty‐five patients had a history of HTN, 22 with CAD, and 12 with PVD. The average NIHSS at admission was 5.7 and 3.6 at discharge. Fifty‐nine patients were on aspirin, 15 on clopidogrel, and 32 were on DAPT at presentation. Stroke classification was divided into 27 patients with small vessel disease, 28 with large vessel disease, 18 with cardiogenic origin, and 33 with cryptogenic. Of this demographic data collected, the only statistically significant difference between the two groups was noted with more cryptogenic stroke in patients with recurrent stroke (Table 1).

**TABLE 1:** Baseline characteristics of the study population.

	Patients with primary stroke	Patients with recurrent stroke	*p*
Overall (*n*=)	52	106	
Age	68	67	0.72
Races (Caucasian, %)	92%	91%	0.98
Gender (male, %)	57%	59%	0.96
Smoking	20	40	0.47
BMI	29	31	0.25
HTN	27	55	0.71
LDL	94	82	0.56
HbA1c	6.4	6.3	0.95
CAD	15	22	0.90
PVD	6	12	0.56
NIHSS at admission	3.5	5.7	0.10
NIHSS at discharge	2.4	3.6	0.35
Aspirin	32	59	0.68
Clopidogrel	8	15	0.46
Aspirin and clopidogrel	12	32	0.58
Stroke subtype			
Small vessel disease	14	27	0.89
Large vessel disease	15	28	0.93
Cardiogenic	10	18	0.85
Cryptogenic	13	33	0.04

Abbreviations: BMI = body mass index, CAD = coronary artery disease, HbA1c = hemoglobin A1c, HTN = hypertension, LDL = low‐density lipoprotein, NIHSS = National Institutes of Health Stroke Scale, PVD = peripheral vascular disease.

In Table 2, we evaluate the results of our VerifyNow assays in patients with primary and recurrent stroke. In the primary stroke group, 44 patients were on aspirin, with 2 (4%) demonstrating inadequate platelet inactivation on testing. Twenty patients with primary stroke were on clopidogrel, with 6 (30%) demonstrating inadequate platelet inactivation. In the recurrent stroke group, 91 patients were on aspirin, with 12 (13%) demonstrating inadequate platelet inactivation. Forty‐seven patients were on clopidogrel, with 18 (38%) demonstrating inadequate platelet inactivation. Patients with recurrent stroke showed a significantly high incidence of nonresponding rate to aspirin (*p* < 0.05) compared to the group of patients with primary stroke. A similar phenomenon was found in patients taking clopidogrel. The incidence of nonresponding rate to clopidogrel is high in patients with recurrent stroke compared to patients with primary stroke, but there is no significant difference.

**TABLE 2:** Evaluation of platelet function by VerifyNow assay.

	Patients with primary stroke	Patients with recurrent stroke
Aspirin VerifyNow		
Overall (*n*=)	44	91
Responders (*n*, %)	42 (96%)	79 (87%)
Noresponders (*n*, %)	2 (4%)	12 (13%)
Clopidogrel VerifyNow assay		
Overall (*n*=)	20	47
Responders (*n*, %)	14 (70%)	29 (62%)
Noresponders (*n*, %)	6 (30%)	18 (38%)

In Table 3, we evaluate the results of VerifyNow assays in Caucasian and non‐Caucasian stroke populations. In the Caucasian group, 123 patients were on aspirin, with 14 (11%) demonstrating inadequate platelet inactivation on testing. Sixty‐one patients were on clopidogrel, with 20 (33%) demonstrating inadequate platelet inactivation. In the non‐Caucasian stroke group, 12 patients were on aspirin, with one demonstrating inadequate platelet inactivation (8%). Six patients were on clopidogrel, with one demonstrating inadequate platelet inactivation (17%). There is a significantly high incidence of non‐response rate to clopidogrel (*p* < 0.05), but not to aspirin, in the Caucasian stroke population.

**TABLE 3:** Evaluation of platelet function by VerifyNow assay, the race difference.

	Caucasian	Non‐caucasian
Aspirin VerifyNow		
Overall (*n*=)	123	12
Responders (*n*, %)	109 (89%)	11 (92%)
Noresponders (*n*, %)	14 (11%)	1 (8%)
Clopidogrel VerifyNow assay		
Overall (*n*=)	61	6
Responders (*n*, %)	41 (67%)	5 (83%)
Noresponders (*n*, %)	20 (33%)	1 (17%)

In Table 4, we evaluate the results of our VerifyNow assays in male and female stroke populations. In the male group, 78 patients were on aspirin, with 13 (17%) demonstrating inadequate platelet inactivation during testing. Thirty‐nine male patients were on clopidogrel, with 5 (13%) demonstrating inadequate inactivation. In the female stroke group, 57 patients were on aspirin, with two demonstrating inadequate platelet inactivation (4%). Twenty‐eight female patients were on clopidogrel, with 10 demonstrating inadequate platelet inactivation (36%). There is a significantly high incidence of non‐response rate to aspirin in males and to clopidogrel in the female stroke population (*p* < 0.05).

**TABLE 4:** Evaluation of platelet function by VerifyNow assay, the gender difference.

	Male	Female
Aspirin VerifyNow		
Overall (*n*=)	78	57
Responders (*n*, %)	65 (83%)	55 (96%)
Noresponders (*n*, %)	13 (17%)	2 (4%)
Clopidogrel VerifyNow assay		
Overall (*n*=)	39	28
Responders (*n*, %)	34 (87%)	18 (64%)
Noresponders (*n*, %)	5 (13%)	10 (36%)

In Table 5, we evaluated the difference between the subsets of ischemic stroke with small vessel or large vessel disease, as these are the two subtypes of ischemic stroke where antiplatelet therapy is the standard regimen in secondary stroke prevention. In the small vessel disease group, 35 patients were on aspirin, with 5 (14%) demonstrating inadequate platelet inactivation, and 18 were on clopidogrel, with 3 (17%) demonstrating inadequate platelet inactivation. Of the patients in the large vessel disease group, 31 were on aspirin, 4 (13%) demonstrated inadequate platelet inactivation, and 21 were on clopidogrel, with 3 (14%) demonstrating inadequate platelet inactivation. There was not a statistically significant difference between these two groups (small vessel disease compared to large vessel disease as patient's stroke mechanism) regarding either aspirin resistance or clopidogrel resistance.

**TABLE 5:** Evaluation of platelet function by VerifyNow assay, the differences among stroke subtypes.

	Small vessel disease	Large vessel disease
Aspirin VerifyNow		
Overall (*n*=)	35	31
Responders (*n*, %)	30 (86%)	27 (87%)
Noresponders (*n*, %)	5 (14%)	4 (13%)
Clopidogrel VerifyNow assay		
Overall (*n*=)	18	21
Responders (*n*, %)	15 (83%)	18 (86%)
Noresponders (*n*, %)	3 (17%)	3 (14%)

## 4 Discussion

As stroke treatments and methods of stroke prevention have developed over the years, several studies have been conducted that have demonstrated the efficacy of utilizing antiplatelet therapy; mainly, DAPT has been widely applied in patients with minor stroke or TIA after the clopidogrel in high‐risk patients with acute nondisabling cerebrovascular events (CHANCE) [14] and the platelet‐oriented inhibition in new TIA and minor ischemic stroke (POINT) trial [15] being published. The American Heart Association (AHA) 2019 update to the 2018 guidelines for early management of acute ischemic stroke recommended DAPT can be started within 24 h after symptom onset and continued for 21 days [8]; the treatment window of this regimen can be extended up to 72 h after stroke onset [16]. Methods to appropriately identify stroke etiology have helped to increase the efficacy of the choice of therapy. Etiologies not related to cardioembolism, including large vessel disease and small vessel disease, show the greatest benefit to antiplatelet therapies, with aspirin often being the first‐line therapy for most patients and clopidogrel being used as an alternative or in combination [17]. Despite compliance with the appropriately chosen therapy, however, there are still patients who continue to have a recurrence of ischemic events. This may be due to incorrect stroke etiology identification, poor risk factor control, or the presence of non‐modifiable risk factors. If the aforementioned factors do not play a role, the question of adequate medication response is raised. Several mechanisms of inadequate response to antiplatelet therapy have been suggested, including medication adherence, drug absorption and metabolism, variation in pharmaceutical preparations, drug interactions, high platelet turnover, environmental/lifestyle factors, and modifications of a drug's therapeutic target [18]. VerifyNow laboratory testing [19] has been developed to help better identify patients with inherent resistance to these medications, which is a cost‐effective platelet function‐tailored strategy with antiplatelet therapy [20]. If a stroke patient has an inadequate response to aspirin, an alternative option can be to switch from aspirin to cilostazol. Cilostazol is a PDE3 (phosphodiesterase III) inhibitor with a long track record of safety that is FDA and European Medicines Agency approved for treating claudication in patients with peripheral arterial disease. Cilostazol appears safe and does not increase the risk of major bleeding when given alone or in combination with aspirin or clopidogrel [21, 22] and has been reported to be effective for the secondary prevention of ischemic stroke [23], particularly in lacunar stroke commonly resulting from small vessel disease [24]. Clopidogrel is a prodrug that needs to be absorbed through the intestine and metabolized by the liver before it can work. Similar to clopidogrel, ticagrelor inhibits adenosine diphosphate (ADP) receptors of subtype P2Y12. However, ticagrelor does not require hepatic activation of CYP2C19 compared to clopidogrel. In the acute stroke or transient ischemic attack treated with ticagrelor and ASA for prevention of stroke and death (THALES) trial [25], patients with a minor or moderate ischemic stroke had consistent benefit from ticagrelor plus aspirin versus aspirin alone compared to patients with less severe ischemic stroke, with no further increase in the risk of intracranial bleeding or other severe systemic bleeding events. That said, if patients do not respond to clopidogrel, ticagrelor can be considered as an alternative monotherapy or DAPT with aspirin.

Our study demonstrated real‐world evidence of resistance to both aspirin and clopidogrel in the subsets of the population based on sex and ethnicity, as well as in patients with recurrent stroke. Notable differences regarding aspirin unresponsiveness are seen at 4% in our female population compared to 17% in males. The opposite was true of clopidogrel resistance, in which the female population demonstrated 36% resistance compared to 13% in the males. Sex differences in aspirin or clopidogrel resistance primarily stem from variations in platelet biology between genders, where women generally exhibit higher baseline platelet reactivity, potentially leading to greater resistance to these antiplatelet medications compared to men; this is often attributed to hormonal factors and differences in the expression of platelet receptors [26, 27]. Estrogen, a female hormone, is thought to play a role in enhancing platelet aggregation, contributing to increased platelet reactivity in women [28]. There was also a low incidence of resistance to aspirin (8%) or clopidogrel (17%) noted in the non‐Caucasian population compared to the Caucasian population, with 11% resistance to aspirin and 33% resistance to clopidogrel. These results further raise the question as to whether there is an increased propensity toward resistance with different antiplatelet agents regarding sex and ethnicity. There are several limitations that should be kept in mind. One limitation of this study is the relatively small sample size, which may limit the generalizability of the results. A future study with a large sample size, including a diverse patient population with stroke, is undoubtedly needed to validate the findings from our practice. Additionally, previous studies have demonstrated a strong association between CYP2C19 genetic polymorphisms and their effect on clopidogrel. However, it is important to note that not all individuals with CYP2C19 gene mutations have clopidogrel resistance [29]. Intermediate metabolizers are able to process some clopidogrel, so they receive partial benefit from the treatment [29]. Due to limitations in insurance coverage, we were unable to routinely screen for CYP2C19 polymorphisms, although ideally, it should be tested to analyze its correlation with clopidogrel resistance.

While these results do suggest that there is a considerable portion of the population that may be resistant to our most used medications in preventing stroke and cardiovascular events, our observational results are limited by a small sample size, with the majority of them being Caucasian. The sample size of this study for non‐Caucasians is quite small, with only 12 patients on aspirin and six on clopidogrel. Therefore, it may not accurately reflect the circumstances in other ethnic groups, and it should be cautious when extending our results to a more diverse population. Additionally, we acknowledge that this was a retrospective study, lacking genetic testing for CYP2C19 polymorphisms, which may limit the generalizability of our findings. Large‐scale studies should be pursued to evaluate these differences further and provide the opportunity for individualized treatment plans and more efficacious stroke prevention for the general population. We should be aware of the limitations of antiplatelet agents and how to approach the evaluation of a stroke patient with a recurrent event despite medication adherence and optimized underlying vascular risk factors.

## Author Contributions

J.M. designed and oversaw the study; S.C. performed research and drafted the manuscript; A.D. analyzed data; L.P., A.A., M.M., N.K., and N.W. contributed to data collection, reviewed, and edited the manuscript; L.P. managed the project. All authors have read and agreed to the published version of the manuscript.

## Conflicts of Interest

The authors declare no conflicts of interest.

## Data Availability

The data that support the findings of this study are available on request from the corresponding author upon reasonable request.
